# Metabolic health phenotypes and cardiovascular disease among U.S. adults with central obesity: Evidence from NHANES 2011– March 2020

**DOI:** 10.1016/j.obpill.2026.100267

**Published:** 2026-04-14

**Authors:** David Kweku Aseda Donkor, Emmanuel Amoako Agyei, Samuel Prince Osei, Frederick Frimpong Opuni, Evelyn Gyau-Boakye, Maxwell Emmie, Adwoa Benyiwah Amoah, Sandra Agyenim Boateng, Dennis Dela Tsagli, Eunice Akomaning

**Affiliations:** a Department of Internal Medicine, Wellstar Spalding Regional Hospital, Griffin, GA, 30224, USA; b Department of Biostatistics, Washington University in St. Louis, St. Louis, MO, 63130, USA; c Department of Internal Medicine, Cape Fear Valley Medical Center, Fayetteville, NC, 28304, USA; d Department of Internal Medicine, Nancy Powell Clinic, Konongo, Tebeso II, Ghana; e Department of Pediatrics, Ga East Municipal Hospital, Kwabenya, WY 1895, Ghana; f Department of Internal Medicine, Sekyere Kumawu District Hospital, Kumawu, B 29, Ghana; g Department of Internal Medicine, Shai-Osudoku District Hospital, Ayikuma Rd, Dodowa, Ghana; h School of Public Health, Washington University in St. Louis, St. Louis, MO, 63130, USA; i Department of Medicine and Therapeutics, Korle-Bu Teaching Hospital, Guggisberg Avenue, Korle Bu, Accra, Ghana

**Keywords:** Abdominal obesity, Cardiovascular diseases, Metabolic syndrome, Risk stratification

## Abstract

**Background:**

Central obesity is strongly associated with cardiometabolic dysfunction and cardiovascular disease (CVD), yet CVD burden may vary by metabolic phenotype among affected adults. This study examined whether metabolically unhealthy central obesity (MUCO) is associated with higher CVD prevalence than metabolically healthy central obesity (MHCO), whether CVD increases in a dose-response manner with metabolic abnormality burden, and whether associations vary by age, sex, and race/ethnicity.

**Methods:**

We conducted a cross-sectional analysis of NHANES 2011–March 2020, including adults ≥20 years with central obesity (≥102 cm (40″) in men; ≥88 cm (35″) in women). Metabolic phenotype was defined by four abnormalities: elevated blood pressure (≥130/85 mmHg or antihypertensive use), dysglycemia (HbA1c ≥ 5.7%, self-reported diabetes, or diabetes medication use), low HDL cholesterol (<40 mg/dL in men or <50 mg/dL in women), and hypercholesterolemia (physician diagnosis or lipid-lowering therapy). Participants were classified as MHCO (0–1 abnormality) or MUCO (≥2 abnormalities); abnormalities were summed (0–4) to assess dose-response. The outcome was self-reported physician-diagnosed CVD (coronary heart disease, myocardial infarction, stroke, heart failure, or angina). Survey-weighted Poisson regression estimated adjusted prevalence ratios (PRs) and 95% confidence intervals (CIs).

**Results:**

Among 11,657 adults with central obesity, 6,997 were MHCO and 4,660 MUCO, representing 128.4 million U.S. adults. Weighted CVD prevalence was higher in MUCO than MHCO (15.7% vs 7.3%). After adjustment, MUCO was associated with higher CVD prevalence (PR 1.48; 95% CI 1.30–1.68; p < 0.001). Each additional metabolic abnormality increased CVD prevalence by 29% (PR 1.29; 95% CI 1.21–1.38; p < 0.001). The association was strongest among adults aged 20–39 years (PR 3.89; 95% CI 2.22–6.81), despite low absolute prevalence.

**Conclusions:**

Among U.S. adults with central obesity, metabolic dysfunction is associated with higher CVD prevalence, with a clear dose-response relationship. These findings support phenotype-based cardiovascular risk stratification beyond waist circumference and emphasize early identification and management of metabolic abnormalities, particularly in younger adults.

## Graphical abstract

**Figure:**
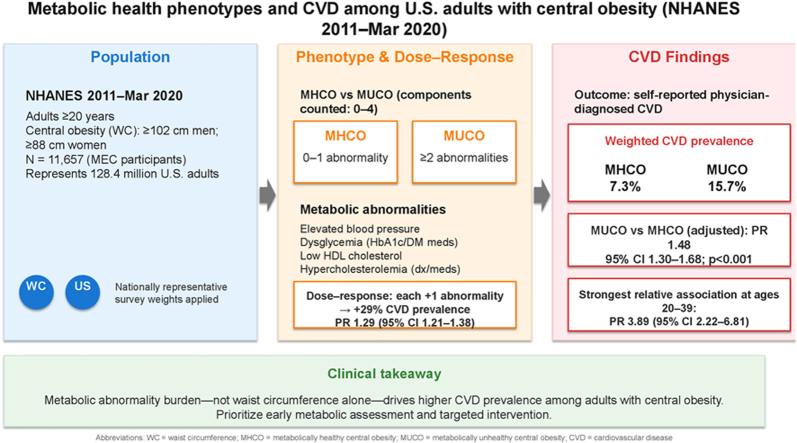

## Abbreviations

ATP III: Adult Treatment Panel III
CI: Confidence Interval
CVD: Cardiovascular Disease
HbA1c: Glycated Hemoglobin
HDL-C: High-Density Lipoprotein Cholesterol
MEC: Mobile Examination Center
MHCO: Metabolically Healthy Central Obesity
MUCO: Metabolically Unhealthy Central Obesity
NHANES: National Health and Nutrition Examination Survey
WC: Waist Circumference

## 1 Introduction

Obesity is a chronic disease defined by excess adiposity and is a leading cause of cardiometabolic morbidity internationally. Global obesity prevalence has significantly increased in the past few decades, and the World Health Organization (WHO) reported that obesity was nearly three times more common worldwide than before 1975 [1]. In the United States, recent NHANES data (August 2021–August 2023) estimate that 40.3% of adults are living with obesity and 9.4% have severe obesity, highlighting the continued public health burden [2]. While obesity is usually associated with insulin resistance, dyslipidemia, high blood pressure, and high cardiovascular risk, a subset of those with obesity have a mildly adverse cardiometabolic profile. This phenotype has been characterized as metabolically healthy obesity (MHO) [3,4]. Nevertheless, the concept has been controversial, as no definition is widely accepted [5,6]. Numerous “MHO” classifications could reflect less complex metabolic illness than actual health, and metabolic health can be transient [7]. Longitudinal evidence summarized in previous work indicates that a significant proportion of those initially labeled MHO reach metabolically unhealthy obesity (MUO) at follow-up, with worsening insulin sensitivity and glycemia often implicated [5]. Central (abdominal) obesity has particular relevance to cardiometabolic risk because it more closely represents visceral adiposity and ectopic fat distribution compared with BMI alone [8]. Cardiovascular disease (CVD) is still the leading cause of death worldwide, and high central obesity is a significant risk factor for CVD [9]. Abdominal obesity is common among U.S. adults, and a higher waist circumference in abdominal obesity is associated with a higher incidence of cardiometabolic diseases and a higher risk for mortality. A focus on categorizing central obesity severity for risk stratification has been recommended [10] based on NHANES analyses. In addition to these national estimates, prospective cohorts' data, which have been pooled and assessed in a dose–response meta-analysis, suggest that increasing waist circumference and associated abdominal obesity indices are linked to the development of CVD, emphasizing the evidence on the clinical relevance of central measures in body fat rather than BMI [11]. However, several important gaps exist in the operationalization and application of metabolic health status in populations with central obesity that contribute to the growing literature on obesity phenotypes. Previous U.S. work has examined temporal trends in metabolically healthy obesity phenotypes (abdominal obesity classifications included). Still, trend-oriented analyses have not been designed to quantify current phenotype-related CVD prevalence associations in a central-obesity–restricted analytic sample [12]. Moreover, although studies of abdominal obesity severity have examined a broad cardiometabolic disease profile and mortality, they do not directly address the comparative burden of self-reported CVD across metabolic health phenotypes defined within the adult population with central obesity, utilizing a simple count of metabolic abnormalities in agreement with a clustering of clinical risk factors [10]. Using pre-pandemic NHANES 2011–March 2020 data, the current study examines the association between metabolic health phenotype among adults with central obesity and prevalent cardiovascular disease (CVD). We estimated the association among metabolically unhealthy central obesity (MUCO) (vs metabolically healthy central obesity [MHCO]) and prevalent CVD with prevalence ratios, assessed a dose–response relationship between the number of metabolic abnormalities and the prevalence of CVD, and assessed the effect modifications for age, sex, and race/ethnicity to inform phenotype-based cardiovascular risk stratification among U.S. adult population with central obesity.

## 2 Methods

### 2.1 Study design and data source

We conducted a cross-sectional analysis using data from the National Health and Nutrition Examination Survey (NHANES) covering survey cycles 2011–2012, 2013–2014, 2015–2016, and 2017–March 2020 (pre-pandemic). NHANES is a nationally representative survey of the non-institutionalized U.S. population that uses a complex, multistage probability sampling design and includes household interviews, standardized physical examinations, and laboratory measurements. All NHANES protocols were approved by the National Center for Health Statistics (NCHS) Research Ethics Review Board, and all participants provided written informed consent. Because this study used publicly available, de-identified data, additional institutional review board approval and informed consent were not required.

### 2.2 Study population

We sampled adults aged ≥20 years who participated in the Mobile Examination Center (MEC) component. Participants were further limited to those with central obesity, defined using waist circumference cut-points from the Adult Treatment Panel III criteria (≥102 cm (40″) for men and ≥88 cm (35″) for women) [13]. These cut-points were selected to align with widely used clinical criteria and to maintain consistency across NHANES cycles. We excluded individuals with missing survey design variables (sampling weights, strata, or primary sampling units). A stepwise flow diagram was used to document exclusions and derive the final analytic sample.

### 2.3 Exposure: metabolic health phenotype

Among adults with central obesity, metabolic health status was defined by the presence of four metabolic abnormalities: elevated blood pressure, dysglycemia, low high-density lipoprotein (HDL) cholesterol, and hypercholesterolemia.

Elevated blood pressure was defined as mean systolic blood pressure ≥130 mmHg, mean diastolic blood pressure ≥85 mmHg, or self-reported use of antihypertensive medication. Dysglycemia was defined as an HbA1c level of ≥5.7%, self-reported diabetes, or the use of diabetes medication. Low HDL cholesterol was defined as <40 mg/dL in men or <50 mg/dL in women. Hypercholesterolemia was defined as a self-reported physician diagnosis or use of lipid-lowering medication. Hypercholesterolemia was included as a lipid abnormality based on the NHANES questionnaire and medication data to capture clinically recognized dyslipidemia. Triglyceride levels were not included in the primary phenotype definition to preserve sample size and avoid restricting analyses to fasting subsamples.

For each participant, the total number of metabolic abnormalities was summed, yielding a score ranging from 0 to 4. Participants were then categorized as metabolically healthy central obesity (MHCO) if they had 0–1 metabolic abnormality, and metabolically unhealthy central obesity (MUCO) if they had ≥2 metabolic abnormalities.

### 2.4 Outcome: cardiovascular disease

The primary outcome was any cardiovascular disease (CVD), defined using self-reported physician diagnoses from the NHANES Medical Conditions Questionnaire. This approach has been used in previous NHANES cardiovascular epidemiology studies and enables consistent ascertainment across cycles. Participants were classified as having CVD if they reported a history of coronary heart disease, myocardial infarction, stroke, heart failure, or angina. A binary indicator was created such that any affirmative response to one or more of these conditions resulted in classification as having CVD**.**

### 2.5 Covariates

Covariates included age (measured continuously in years), sex (male/female), and race/ethnicity, categorized according to NHANES definitions. Non-Hispanic White was specified as the reference category in all regression models. These covariates were selected a priori based on their established associations with both metabolic health and cardiovascular disease.

### 2.6 Survey weighting and pooled design

All analyses incorporated NHANES sampling weights, strata, and primary sampling units to account for the complex survey design and produce nationally representative estimates. Mobile Examination Center (MEC) examination weights were pooled across 4 survey cycles by dividing the 2-year MEC weight by the number of included cycles. Survey design objects were created before all descriptive and regression analyses.

### 2.7 Statistical analysis

Descriptive characteristics were summarized using survey-weighted means and standard errors for continuous variables and survey-weighted percentages for categorical variables, stratified by metabolic phenotype. Associations between metabolic phenotype and CVD prevalence were estimated using survey-weighted generalized linear models with a Poisson distribution and log link, yielding prevalence ratios (PRs) and 95% confidence intervals (CIs). This approach was selected because the outcome prevalence was not rare, and prevalence ratios provide more interpretable estimates than odds ratios in cross-sectional studies.

Three primary modeling objectives were evaluated:

Overall association between MUCO (vs MHCO) and CVD prevalence.

Dose–response relationship between the number of metabolic abnormalities and CVD prevalence. Additional exploratory analysis evaluating the independent associations of individual metabolic abnormalities with prevalent CVD overall and among younger adults (20–39 years) was done.

Effect modification by age group, sex, and race/ethnicity was assessed using interaction terms.

Age-specific prevalence ratios were derived from interaction models, and model-based predicted CVD prevalence was estimated across age groups for each phenotype while holding sex and race/ethnicity constant at reference values. We visualized results using forest plots and predicted prevalence curves. All analyses were conducted using R (survey and srvyr packages). Statistical significance was assessed at a two-sided α level of 0.05.

### 2.8 Sensitivity analysis

Sensitivity analyses included: (1) redefining metabolic health using a stricter phenotype definition (0 vs ≥ 1 metabolic abnormalities), (2) additional adjustment for smoking history (ever vs never smoking) to assess potential confounding by tobacco exposure, (3) redefining dysglycemia using only HbA1c ≥ 5.7% or self-reported diabetes, excluding medication use from the definition to address potential misclassification arising from diabetes medication use for non-glycemic indications (e.g., metformin for polycystic ovary syndrome or GLP-1 receptor agonists for weight management).

### 2.9 Data availability

The data analyzed in this study are publicly available from the National Health and Nutrition Examination Survey (NHANES), conducted by the U.S. Centers for Disease Control and Prevention (CDC). No new data were generated for this study. Analytic code and derived variables supporting the findings of this study are available from the corresponding author upon reasonable request.

## 3 Results

### 3.1 Study population characteristics

Among adults with central obesity included in the analytic sample (unweighted N = 11,657), 6,997 were classified as metabolically healthy central obesity (MHCO) and 4,660 as metabolically unhealthy central obesity (MUCO) (Table 1). When survey weights were applied, this analytic sample represented approximately 128.4 million U.S. adults with central obesity during 2011 to March 2020, including an estimated 84.7 million classified as MHCO and 43.7 million classified as MUCO.

**Table 1:** Survey-weighted characteristics of adults with central obesity by metabolic phenotype (NHANES 2011–March 2020).

Characteristic	MHCO (n = 6,997)^a^	MUCO (n = 4,660)^a^
Weighted population, millions	84.7 million	43.7 million

*Mean (SE)*		

Age (years)	47.7 (0.3)	55.9 (0.3)
Waist circumference (cm) [inches]	107.5[42″] (0.2)	113.9[45″] (0.3)
HbA1c (%)	5.5 (0.0)	6.4 (0.0)
HDL cholesterol (mg/dL)	55.1 (0.4)	44.0 (0.3)
Metabolic abnormalities count	0.6 (0.0)	2.2 (0.0)
Any CVD, % (SE)	7.3% (0.4%)	15.7% (0.8%)

*Percentage % (within)*		

Sex %:		
Male, %	37.8%	43.2%
Female, %	62.2%	56.8%
Race/ethnicity %:		
Non-Hispanic White, %	68.7%	63.4%
Mexican American, %	9.1%	9.4%
Non-Hispanic Asian, %	2.2%	3.3%
Non-Hispanic Black, %	10.6%	13.9%
Other Hispanic, %	5.7%	6.3%
Other Race - Including Multi- Racial %	3.6%	3.7%

Values are survey-weighted estimates. Continuous variables are shown as mean (SE). Binary variables are shown as percent (SE).

CVD = Cardiovascular Disease.

HbA1c = Glycated Hemoglobin.

HDL-C = High-Density Lipoprotein Cholesterol.

MHCO = Metabolically Healthy Central Obesity.

MUCO = Metabolically Unhealthy Central Obesity.

NHANES = National Health and Nutrition Examination Survey.

SE = Standard Error.

a _ unweighted sample sizes.

Participants with MUCO were older on average than those with MHCO (mean age 55.9 vs 47.7 years) and had greater waist circumference (113.9 vs 107.5 cm) (45″ vs 42″). Adults classified as MUCO exhibited worse metabolic profiles, including higher mean HbA1c (6.4% vs 5.5%), lower HDL cholesterol (44.0 vs 55.1 mg/dL), and, as expected given the phenotype definitions, MUCO had a higher mean abnormality count than MHCO (2.2 vs 0.6), supporting appropriate phenotype classification.

**Fig. 1:**
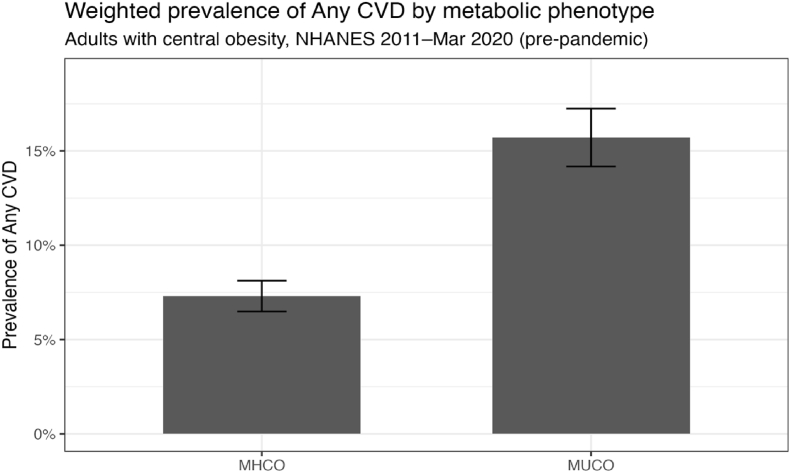
Weighted prevalence of any CVD by metabolic phenotype CVD = Cardiovascular Disease MHCO = Metabolically Healthy Central Obesity MUCO = Metabolically Unhealthy Central Obesity NHANES = National Health and Nutrition Examination Survey.

The survey-weighted prevalence of any cardiovascular disease (CVD) was more than twice as high among MUCO compared with MHCO (15.7% vs 7.3%) (Fig. 1). Sex distribution differed modestly by phenotype (MUCO 56.8% female; MHCO 62.2% female). Racial and ethnic composition differed modestly: MUCO included a lower proportion of non-Hispanic White participants (63.4% vs 68.7%) and a higher proportion of non-Hispanic Black participants (13.9% vs 10.6%), while other racial and ethnic groups were comparably represented.

### 3.2 Association between metabolic phenotype and cardiovascular disease

In adjusted survey-weighted Poisson regression models (Table 2a), MUCO was associated with a significantly higher prevalence of any CVD compared with MHCO (prevalence ratio [PR] = 1.48; 95% CI: 1.30 to 1.68; p < 0.001), after adjustment for age, sex, and race/ethnicity. Age was strongly associated with CVD prevalence (PR = 1.06 per year; 95% CI: 1.05 to 1.06), and Female participants had lower CVD prevalence compared with male participants (PR = 0.73; 95% CI: 0.63 to 0.85).

**Table 2a:** Adjusted prevalence ratios for any CVD by metabolic phenotype (MUCO vs MHCO).

Predictor	PR (95% CI)	p-value
Phenotype: MUCO (ref: MHCO)	1.48 (1.30–1.68)	<0.001
Age	1.06 (1.05–1.06)	<0.001
Sex: Female (ref: Male)	0.73 (0.63–0.85)	<0.001
Race/ethnicity:		
Mexican American	0.83 (0.67–1.02)	0.084
Non-Hispanic Asian	0.72 (0.49–1.05)	0.097
Non-Hispanic Black	1.22 (1.05–1.42)	0.011
Other Hispanic	0.93 (0.75–1.15)	0.491
Other Race - Including Multi-Racial	1.92 (1.36–2.72)	<0.001

Survey-weighted Poisson regression with log link. PRs adjusted for age, sex, and race/ethnicity. Unweighted N = 11,657. NHANES 2011–March 2020 (pre-pandemic); adults with central obesity.

MHCO = Metabolically Healthy Central Obesity.

MUCO = Metabolically Unhealthy Central Obesity.

NHANES = National Health and Nutrition Examination Survey.

PR = Prevalence ratio.

Using non-Hispanic White participants as the reference group, non-Hispanic Black participants had a higher prevalence of CVD (PR = 1.22; 95% CI: 1.05 to 1.42), as did participants categorized as Other Race (including multiracial) (PR = 1.92; 95% CI: 1.36 to 2.72). Differences for Mexican American, non-Hispanic Asian, and other Hispanic participants were not statistically significant.

### 3.3 Dose–response relationship between metabolic abnormality burden and cardiovascular disease

A strong dose–response relationship was observed between metabolic abnormality burden and CVD prevalence (Table 2b). In adjusted models, each additional metabolic abnormality was associated with a 29% higher prevalence of any CVD (PR = 1.29 per +1 abnormality; 95% CI: 1.21 to 1.38; p < 0.001), independent of age, sex, and race/ethnicity. Associations for age, sex, and race/ethnicity were consistent with those observed in the phenotype-based model.

**Table 2b:** Adjusted prevalence ratios for Any CVD by metabolic abnormality burden.

Predictor	PR (95% CI)	p-value
Metabolic abnormalities (per +1)	1.29 (1.21–1.38)	<0.001
Age (per 1 year)	1.06 (1.05–1.06)	<0.001
Sex: Female (ref: Male)	0.74 (0.64–0.85)	<0.001
Race/ethnicity		
Mexican American	0.81 (0.65–1.01)	0.064
Non-Hispanic Asian	0.71 (0.49–1.04)	0.083
Non-Hispanic Black	1.20 (1.03–1.40)	0.020
Other Hispanic	0.91 (0.73–1.14)	0.417
Other Race - Including Multi-Racial	1.91 (1.34–2.71)	<0.001

Survey-weighted Poisson regression with log link. PR represents a change in CVD prevalence per +1 metabolic abnormality. Adjusted for age, sex, and race/ethnicity. Unweighted N = 11657. NHANES 2011–March 2020 (pre-pandemic); adults with central obesity.

PR = Prevalence ratio.

**Fig. 2:**
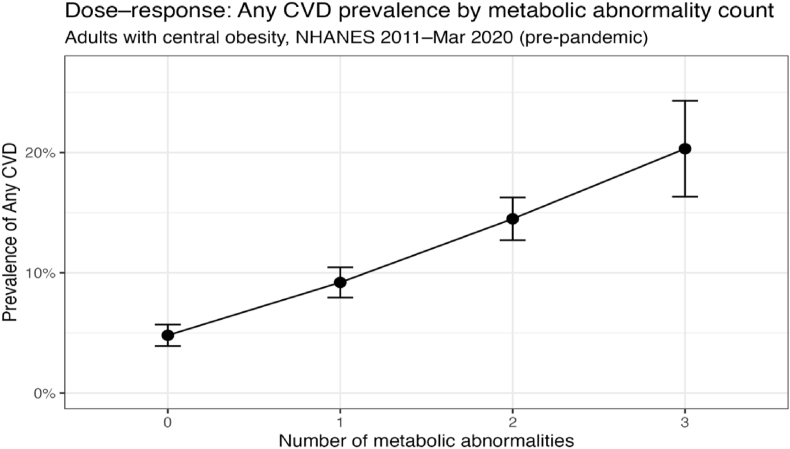
Dose–response relationship between metabolic abnormality counts and any CVD prevalence CVD = Cardiovascular Disease.

Descriptively, CVD prevalence increased monotonically with the number of metabolic abnormalities, rising from approximately 5% among those with no abnormalities to over 20% among those with three abnormalities (Fig. 2), supporting a graded relationship between cumulative metabolic dysfunction and cardiovascular burden. In an exploratory analysis examining individual metabolic abnormalities simultaneously, hypercholesterolemia (PR 2.02; 95% CI 1.72–2.37) demonstrated the strongest association with prevalent CVD, followed by dysglycemia (PR 1.64; 95% CI 1.40–1.92) and low HDL cholesterol (Supplemental Table 1).

### 3.4 Age-specific associations and effect modification

**Fig. 3:**
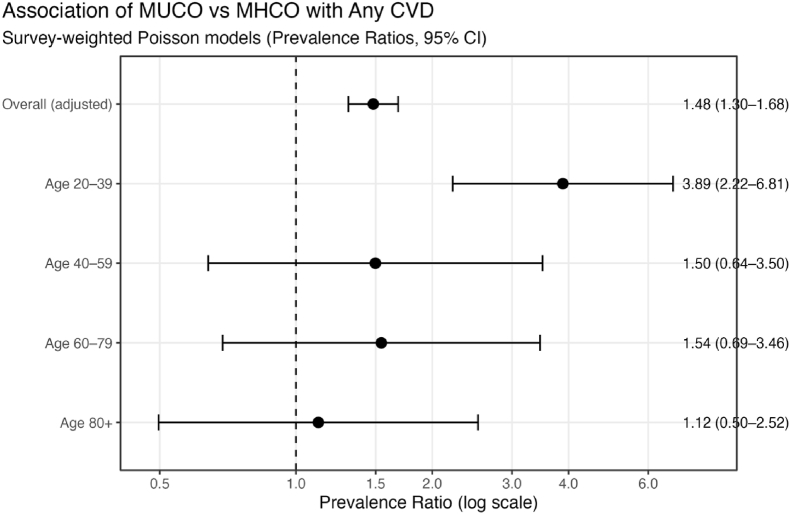
Association of MUCO vs MHCO with any CVD (Forest plot) CVD = Cardiovascular Disease MHCO = Metabolically Healthy Central Obesity MUCO = Metabolically Unhealthy Central Obesity NHANES = National Health and Nutrition Examination Survey.

Age-stratified analyses demonstrated heterogeneity in the association between metabolic phenotype and CVD (Fig. 3). The relative association between MUCO and CVD was strongest among younger adults aged 20–39 years (PR = 3.89; 95% CI: 2.22 to 6.81), indicating approximately fourfold higher prevalence of CVD among MUCO compared with MHCO in this age group. However, because the absolute prevalence of CVD was low in this age group, these relative estimates should be interpreted with caution. In contrast, associations were weaker and less precise in older age groups, with confidence intervals crossing unity among adults aged 40 years and older. In age-stratified analyses restricted to adults aged 20–39 years with central obesity, dysglycemia (PR 2.39; 95% CI 1.26–4.53) and low HDL cholesterol (PR 2.44; 95% CI 1.26–4.73) were the metabolic abnormalities most strongly associated with the prevalent CVD (Supplemental Table 1). Elevated blood pressure (PR 1.54; 95% CI 0.79–3.01) was associated with increased prevalence of CVD within this age group but was not statistically significant after adjustment.

### 3.5 Model-based predicted prevalence of cardiovascular disease by age and phenotype

**Fig. 4:**
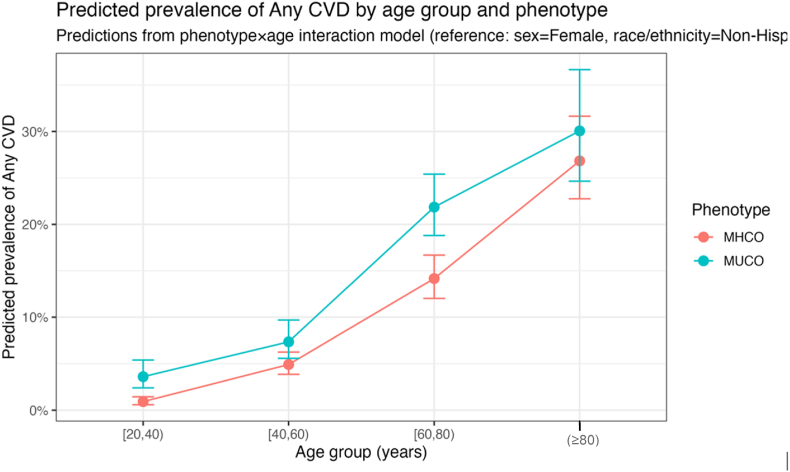
Predicted prevalence of any CVD by age group and metabolic phenotype MHCO = Metabolically Healthy Central Obesity MUCO = Metabolically Unhealthy Central Obesity.

Estimated marginal prevalence models further illustrated the joint effects of age and metabolic phenotype on CVD burden (Table 3; Fig. 4). At reference values of Female sex and non-Hispanic White race/ethnicity, predicted CVD prevalence increased sharply with age in both phenotypes. Across all age groups, MUCO consistently exhibited higher predicted prevalence than MHCO. These values represent model-based comparisons at fixed covariate levels and are intended for illustration rather than population averages.

**Table 3:** Estimated marginal prevalence of any CVD by age group and metabolic phenotype (NHANES 2011–Mar 2020).

Age group (years)	MHCO	MUCO predicted prevalence
	predicted prevalence: % (95% CI)	
20–39	0.9% (0.6–1.4)	3.6% (2.4–5.4)
40–59	4.9% (3.9–6.2)	7.4% (5.6–9.7)
60–79	14.2% (12.0–16.7)	21.9% (18.8–25.4)
≥80	26.8% (22.7–31.6)	30.1% (24.6–36.7)

Predicted prevalence values are model-based estimates from the phenotype × age interaction model, holding sex fixed at Female and race/ethnicity fixed at Non-Hispanic White.

CI = Confidence Interval.

MHCO = Metabolically Healthy Central Obesity.

MUCO = Metabolically Unhealthy Central Obesity.

NHANES = National Health and Nutrition Examination Survey.

In young adulthood (ages 20 to 39), predicted CVD prevalence was low in absolute terms but higher for MUCO than MHCO (3.6% vs 0.9%). With advancing age, both overall CVD prevalence and absolute differences between phenotypes increased. The largest absolute difference was observed among adults aged 60–79 years (21.9% in MUCO vs 14.2% in MHCO), while predicted prevalence reached 26.8% (MHCO) and 30.1% (MUCO) among adults aged 80 years and older.

Importantly, these marginal estimates demonstrate that although relative differences between MUCO and MHCO are greatest at younger ages, when baseline CVD prevalence is low, absolute differences widen with increasing age as overall CVD burden rises. Together, these findings highlight a persistent excess cardiovascular burden associated with greater metabolic abnormality burden across the adult life course, independent of sex and race/ethnicity.

### 3.6 Sensitivity analysis

In a sensitivity analysis using a stricter phenotype definition, where metabolically healthy central obesity was restricted to participants with 0 metabolic abnormalities and the comparison group included those with ≥1 abnormality, the association between metabolic status and prevalent CVD remained significant and was slightly stronger than in the primary analysis (adjusted PR 1.62; 95% CI 1.31–1.99). Additional adjustment for smoking history did not materially alter the association between metabolic phenotype and CVD (Supplemental Table 1). In sensitivity analyses redefining dysglycemia without inclusion of diabetes medication use, the association between metabolically unhealthy central obesity and prevalent CVD remained significant and was slightly stronger (PR 2.34; 95% CI 1.90–2.87). Similarly, each additional metabolic abnormality remained associated with higher CVD prevalence (PR 1.43; 95% CI 1.34–1.53).

## 4 Discussion

The present study was designed to evaluate whether metabolic health phenotype modifies cardiovascular disease (CVD) prevalence among U.S. adults with central obesity and to assess whether increasing metabolic abnormality burden is associated with a graded increase in CVD risk. The study examined metabolic health status and cardiovascular disease among US adults with central obesity between 2011 and March 2020 using a nationally representative sample. An important finding of this study is that we detected a strong dose-response relationship between metabolic abnormality burden and CVD prevalence, even after adjusting for age, sex, and race/ethnicity. To our knowledge, relatively few studies have evaluated a dose-response relationship between these two composite factors. Another important finding of this study is that the relative association between MUCO and CVD was strongest among younger adults aged 20–39 years; however, absolute CVD prevalence in this age group remained low, indicating that the higher relative estimates reflect a greater proportional difference between phenotypes rather than a high overall disease burden. In this age group, MUCO was associated with nearly fourfold higher CVD prevalence than MHCO. In the CARDIA study (Coronary Artery Risk Development in Young Adults), for instance, obesity in young adulthood, irrespective of metabolic status, was linked to early manifestations of cardiac dysfunction, such as left ventricular hypertrophy, and impaired systolic and diastolic function, which are predictive of future CVD [14]. These findings are consistent with the hypothesis that risk processes underlying cardiovascular disease start early in the life course of individuals within the MUCO category [15]. Additionally, a study using coronary artery calcium scoring to identify subclinical ASCVD in adults aged 18–30 years without clinical ASCVD found that individuals with MUCO had greater odds of coronary artery calcium compared with those with MHCO, independent of traditional risk factors [16]. We also found that among younger adults, dysglycemia and low HDL cholesterol emerged as the metabolic abnormalities most strongly associated with CVD prevalence, suggesting that early cardiometabolic risk in centrally obese individuals may be driven more by metabolic dysfunction than by hypertension at younger ages. Our findings add to the growing body of literature that shows the importance of early metabolic abnormalities on future CVD risk, particularly given the increasing prevalence of childhood obesity. This highlights an important time period for early detection and intervention. In addition to the above, our study found that with advancing age, the overall CVD prevalence for both metabolic phenotypes increased. This correlates with progressive vascular aging, cumulative lifetime exposure to several risk factors, and the accumulation of subclinical atherosclerosis [17].

We also found that one-third of individuals fell within the MUCO category. Individuals classified as MUCO had substantially worse cardiometabolic profiles with more than twice the prevalence of CVD compared with the MHCO category. The MUCO category was significantly older, had a larger waist circumference, more pronounced cardiometabolic factors, such as higher HbA1C and lower HDL cholesterol. A report by Gao et al. also reported similar findings [18]. In our study, metabolic health status remains a strong and statistically significant predictor of CVD among the US adult population with central obesity. It is also reflective of how increased visceral obesity, insulin resistance, and dyslipidemia contribute to the development of atherosclerosis and cardiovascular damage [[19], [20], [21]]. From a clinical perspective, these findings suggest that phenotype-based risk stratification may help identify individuals with central obesity who are at disproportionately higher cardiovascular risk despite similar anthropometric profiles, thereby supporting earlier and more targeted risk factor modification and preventive strategies.

Among individual metabolic abnormalities, hypercholesterolemia showed the strongest independent association with prevalent CVD, followed by dysglycemia and low HDL cholesterol. These findings are consistent with established evidence identifying lipid and glycemic dysregulation as major drivers of cardiovascular risk in obesity-related cardiometabolic disease [[19], [20], [21]]. They further suggest that heterogeneity within central obesity phenotypes may be partly explained by differences in lipid and glucose metabolism rather than adiposity alone [[19], [20], [21]].

Differences by race and ethnicity in metabolic health and CVD have received continuous attention in recent years. In line with previous reports, our study found that non-Hispanic Black adults and other racial groups were more likely to be classified as the MUCO group than any of the other groups. These two groups also had a higher prevalence of CVD compared to the non-Hispanic White participants [22,23]. These differences likely reflect a combination of structural determinants of health, differential access to preventive care, and cumulative exposure to cardiometabolic risk factors [24]. These findings underscore the importance of prevention strategies that promote health equity.

### 4.1 Strengths and limitations

#### 4.1.1 Strengths

This study has several strengths. First, data were obtained from NHANES 2011–March 2020, a large, well-designed, and nationally representative survey. This strengthens the validity of our results and enhances generalizability to adults living in the United States with central obesity. In addition, we incorporated the complex survey weights to obtain unbiased population-level estimates and proper standard error estimation. Second, our study's definition of obesity is based on central obesity, measured by waist circumference, rather than BMI. Central adiposity is more reflective of visceral adipose tissue mass, which is known to be more strongly associated with cardiometabolic dysfunction and risk of CVD than overall adiposity. Third, metabolic health was defined based on multiple cardiometabolic components. This allowed us to categorize participants into clinically relevant phenotypes (MHCO vs MUCO) and examine dose–response associations between metabolic abnormality burden and CVD prevalence. Using these metrics, we were also able to move beyond dichotomous definitions of obesity to understand how varying degrees of metabolic dysfunction change cardiovascular risk among adults with central obesity. Fourth, by presenting age-stratified and marginal prevalence estimates, we were able to understand relative and absolute risk patterns across the life course. These estimates show important heterogeneity that can be masked when aggregating participants across all age groups. This approach strengthens the interpretation of age-specific patterns by showing how metabolic health status can drive disproportionate effects at younger ages and larger absolute disease burden as age advances.

#### 4.1.2 Limitations

Limitations of our study include several factors. NHANES’ cross-sectional nature does not allow causal inference and does not allow us to determine whether central obesity precedes the CVD or changes in metabolic health status. As such, it is possible that reverse causality contributed to our findings (i.e., having established CVD could have affected metabolic health or health behaviors). Cardiovascular events were self-reported and subject to recall bias or misclassification. Metabolic health was defined at one point in time and may not account for previous metabolic health states. As metabolic health can change over time, some participants who were classified as MHCO may have experienced metabolic abnormalities in the past. Metabolic abnormalities in the past could place individuals at risk for developing CVD, and our study may underestimate CVD risk in these individuals. It is important to understand that this study reflects CVD prevalence rather than incident disease. We cannot rule out residual confounding by unmeasured factors, such as diet quality, physical activity levels, psychosocial stress, medication adherence, and genetic predisposition. It is therefore crucial to acknowledge the potential impact of unmeasured variables such as physical activity, and economic factors, which could influence both metabolic health and CVD risk. Waist circumference thresholds may differ across populations (e.g., Asian individuals), and the use of uniform ATP III cutoffs may underestimate risk in certain groups. Another key limitation of this study involves the deviation from the traditional definition of metabolic syndrome as proposed by the American Heart Association. Our definition included hypercholesterolemia instead of elevated triglycerides as a core component. This modification was driven by the structure of the NHANES dataset, where triglyceride measurements are limited to fasting participants. Restricting analyses to fasting samples would have reduced the sample size and potentially affected representativeness, thereby limiting generalizability and statistical power. However, this approach differs from standard metabolic syndrome definitions and may limit direct comparability with studies that incorporate triglyceride-based criteria.

### 4.2 Future directions

While research with cross-sectional studies is essential, future studies should involve longitudinal analyses to assess the temporal association between central obesity, metabolic health trajectories, and the development of CVD. The serial measurement of waist circumference and metabolic factors would allow researchers to group individuals based on transitions between MHCO and MUCO states throughout follow-up. Modeling time-updated metabolic health status would allow for better estimation of total and chronic effects of metabolic status on CVD risk. Additionally, outcome ascertainment involving cardiovascular disease should include objective assessments when possible (e.g., stroke, heart failure, or CVD death). Inclusion of subclinical markers of disease (e.g., coronary artery calcium scoring, cardiac magnetic resonance imaging) may also help determine if cardiovascular injury is occurring early in individuals who are young and metabolically unhealthy with central obesity, as they appear to have the greatest relative risk of disease despite low event rates. The evaluation of biological pathways such as inflammation, insulin resistance, adipokines, ectopic fat deposition, and other intermediate phenotypes may further elucidate mechanisms underlying excess CVD associated with greater metabolic abnormality burden. Finally, future studies should assess structural, psychosocial, and environmental contributors to metabolic health status, such as chronic stress, neighborhood factors, health care access, and treatment effect modification, given observed differences in metabolic phenotype across racial and ethnic groups.

## 5 Conclusion

Metabolic health status was strongly associated with cardiovascular disease prevalence in this nationally representative sample of U.S. adults with central obesity. Compared with adults classified as having metabolically healthy central obesity, those classified as having metabolically unhealthy central obesity had consistently higher prevalence of cardiovascular disease across age groups. Differences between metabolic phenotypes were most pronounced in younger adults, although the overall burden of cardiovascular disease was greater at older ages. These findings underscore that central obesity represents a heterogeneous clinical phenotype and suggest that clustered metabolic abnormalities, beyond waist circumference alone, contribute meaningfully to cardiovascular risk. Prevention and management of metabolic abnormalities among adults with central obesity may help reduce overall cardiovascular disease burden.

• Do not assume uniform risk in adults with central obesity. Patients classified as having metabolically unhealthy central obesity (MUCO) had substantially higher cardiovascular disease prevalence than those with metabolically healthy central obesity (MHCO), highlighting clinically relevant heterogeneity.
• Assess cumulative metabolic burden, not waist circumference alone. Each additional metabolic abnormality was associated with a 29% higher prevalence of cardiovascular disease, supporting comprehensive metabolic evaluation in adults with central obesity.
• Prioritize early metabolic risk assessment in younger adults. Although absolute cardiovascular disease prevalence was lower at younger ages, the relative excess risk associated with adverse metabolic profiles was greatest in this group, supporting earlier identification and targeted intervention.

## Author contribution

DKAD contributed to conceptualization, methodology, validation, investigation, data curation, visualization, supervision, project administration, wrote the original draft of the manuscript, and participated in review and editing. EAA contributed to methodology, formal analysis, software, validation, data curation, and review and editing. SPO, FFO, EG, ME, and ABA contributed to the investigation, review, and editing. SAB and DDT contributed to validation, review, and editing. EA contributed to supervision, review, and editing. All authors reviewed and approved the final manuscript before submission.

## Ethical adherence and ethical review

This study used publicly available, de-identified data from the National Health and Nutrition Examination Survey (NHANES), conducted by the National Center for Health Statistics (NCHS), Centers for Disease Control and Prevention. All NHANES protocols were approved by the NCHS Research Ethics Review Board, and written informed consent was obtained from all participants before participation. Because the present study involved secondary analysis of existing, fully de-identified publicly available data and did not involve direct interaction with human subjects, additional institutional review board approval was not required.

## Declaration of the use of artificial intelligence

No artificial intelligence (AI) tools were used in the preparation of this manuscript.

## Funding support

This work received no specific grant from any funding agency in the public, commercial, or not-for-profit sectors.

## Declaration of competing interests

The authors have no financial conflicts of interest, commercial affiliations, or industry relationships relevant to this manuscript.
