# Supplementary material for: Metabolic health phenotypes and cardiovascular disease among U.S. adults with central obesity: Evidence from NHANES 2011– March 2020
Source: Obes Pillars. 2026 Apr 14;18:100267. doi: 10.1016/j.obpill.2026.100267 (PMC13099487; doi:10.1016/j.obpill.2026.100267)
Supplement: Multimedia component 1 [file mmc1.docx]

## Supplemental Table 1. Sensitivity and Exploratory Analyses for Associations Between Metabolic Phenotype and Prevalent Cardiovascular Disease

| **Analysis** | **Exposure** | **Prevalence Ratio (95% CI)** |
| --- | --- | --- |
| Individual metabolic abnormalities and CVD (Overall) | Elevated blood pressure | 0.91 (0.80–1.04) |
|  | Dysglycemia | 1.64 (1.40–1.92) ** |
|  | Low HDL cholesterol | 1.41 (1.25–1.59) ** |
|  | Hypercholesterolemia | 2.02 (1.72–2.37) ** |
| Individual metabolic abnormalities and CVD  (age 20–39 years) | Elevated blood pressure | 1.54 (0.79–3.01) |
|  | Dysglycemia | 2.39 (1.26–4.53) ** |
|  | Low HDL cholesterol | 2.44 (1.26–4.73) ** |
|  | Hypercholesterolemia | 1.88 (0.98–3.59) ** |
| Smoking-adjusted model | MUCO vs MHCO (smoking-adjusted) | 1.47 (1.30–1.66) ** |
| Dysglycemia redefinition model | MUCO vs MHCO | 2.34 (1.90–2.87) ** |

**statistically significant (p<0.05)
